# H_2_(g) production from dimethylamine borane by Cu^0^/WO_3_ NPs catalyst

**DOI:** 10.55730/1300-0527.3550

**Published:** 2023-02-21

**Authors:** Doaa AL-HAMEEDAWI, Seda KARABOĞA, İzzet Amour MORKAN

**Affiliations:** Department of Chemistry, Bolu Abant İzzet Baysal University, Bolu, Turkey

**Keywords:** H_2_ (g) generation, dimethylamine borane, Cu^0^ nanoparticles, tungsten(VI) oxide, heterogeneous catalysis

## Abstract

Cu^0^ NPs supported on tungsten (VI) oxide (WO_3_) were in situ generated from the reduction of Cu^2+^ ions during dehydrogenation of dimethylamine borane (DMAB). The Cu^0^/WO_3_ NPs displayed tangible catalytic activity in H_2_ (g) releasing reaction and they were identified by using advanced techniques. Cu^0^/WO_3_ NPs were found as active catalyst providing one equiv. H_2_(g) per mole of DMAB. The results from TEM images display the formation of Cu^0^ NPs with an average particle size of 4.6 ± 1.0 nm on the surface of WO_3_. Moreover, Cu^0^/WO_3_ NPs with various metal loadings were prepared and tested as catalyst in dehydrogenation reaction to find the optimum metal loading on WO_3_ support. The highest H_2_ generation rate was achieved for 4.0% wt. Cu^0^/WO_3_ catalyst with TOF value of 39 h^−1^ in the reaction conditions. Additionally, effect of various catalyst concentration and temperature is discussed on the reaction kinetics for reaction catalyzed by Cu^0^/WO_3_ NPs.

## 1. Introduction

In the next century, global innovation is necessary for sustainable energy due to peripheral damage by burning fossil fuels [1]. In this regard, the development of renewable energy sources is considered urgent to facilitate the transition from fossil fuel. [2]. Hydrogen is a clean, safe, and sustainable energy carrier that can be used to overcome this problem. [3]. However, the problem of finding safe hydrogen storage materials is the main issue in using H_2_ for mobile and stationary fuel cell applications [4,5]. Borane nitrogen compounds have attracted more attention recently due to their high hydrogen storage capacity and stability [6–7]. Dimethylamine borane (DMAB), one of the B-N adducts, could be used as a solid hydrogen storage material [8–9]. More importantly, (CH_3_)_2_NHBH_3_) is stable and low-cost for regeneration, which could release hydrogen gas with yields up to precisely 3.0 equiv. per mole of DMAB by either through hydrolysis [10,11] or 1.0 equiv. per mole of DMAB from dehydrogenation with an appropriate catalyst under ambient conditions Eq. (1) [12].


(1) 

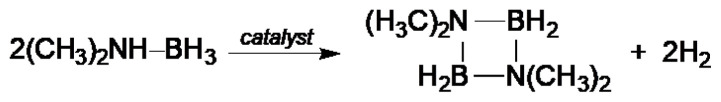


Transition metals could be used as a catalyst in H_2_ (g) releasing reactions of borane adducts [13,14]. So far, homogeneous [10,15–16] and heterogeneous metal nanoparticles [17–18] have been trialed for dehydrogenation of DMAB. Among these metal nanoparticles, most of them were noble metals such as ruthenium [19–20], rhodium [21], and palladium [22]. They are more expensive than nonnoble metals. However, some of the nonnoble metals are suitable as a catalyst in H_2_ releasing reactions such as copper, nickel [23,24]. Cu^0^ NPs have attracted a lot of interest because copper is abundant on the earth and relatively inexpensive among these metals, and it has been used for various purposes in versatile applications and research [25]. The main problem of the Cu^0^ NPs is related with the agglomeration of them during the catalytic reaction. The agglomeration of Cu^0^ NPs lead to the composition of the metal bulk, which causes a fast losing to catalytic activity as expected. In this case, it is necessary to find convenient supporting supplies with huge surface area to prevent the aggregation of transition metal NPs [26]. WO_3_ can be used as a proper support for metal NPs and prevents the formation of bulk metal [27]. So far, WO_3_ has been approved as an effective catalyst for various reactions such as hydrolysis of ammonia borane [28], dehydrogenation of 2-butanol [29], oxidation of ethanol, and methanol [30,31]. In this report, Cu^0^ NPs were stabilized by WO_3_ powder due to reducible nature of WO_3_ supporting material. WO_3_ can facilitate transfer of electrons under reaction conditions and this status causes surplus charge on the surface. The reducible property of WO_3_ improves interaction between the metal and oxide support which is directly related with the catalytic performance of the catalyst. [32]. This advantage makes it a unique supporting material for the metal nanoparticles in several catalytic reactions. Herein, WO_3_ supported Cu^0^ NPs prepared by impregnation of Cu^2+^ ions on powder supporting material in toluene solution. The active catalyst called Cu^0^/WO_3_ NPs was tested as a catalyst in dehydrogenation of DMAB at 60 ± 0.5 °C. The isolated catalyst (Cu^0^/WO_3_ NPs) was identified by modern analytical techniques such as XRD, XPS, UV-Vis, TEM. The results of the experiments and analysis reveal that Cu^0^ NPs are an active catalyst and they provide (39 h^−1^) initial TOF in dehydrogenation reaction of DMAB at 60 ± 0.5 °C. Additionally, the report encloses the evaluation of kinetic studies of the catalytic reaction of DMAB.

## 2. Experimental

### 2.1 Materials

Tungsten trioxide (WO_3_, 99.99%) was purchased from Nanografi. Toluene, copper bis(2-ethylhexanoate), and dimethylamine borane (97%) were bought from Sigma Aldrich. Toluene used as a solvent in this study was purified before performing the catalytic experiments. All glassware was cleaned with ethanol and dried at 120 °C.

### 2.2. Instrumentation

The same instruments have been utilized as one given our previous work, except for TEM and UV-Vis analysis [31]. While the TEM images of the Cu^0^/WO_3_ NPs were obtained from Hitachi HT-7700 operating at 120 kV, UV-Vis spectra of initial copper precursor Cu(II) 2-ethylhexanoate) and final catalyst Cu^0^ NPs were taken by HITACHI U-2900 UV/VIS spectrophotometer.

### 2.3. Determination of the most effective Cu loading for Cu^0^/WO_3_ NPs

Different copper loaded samples (1.0%–6.0% wt.) were prepared in order to determine the most effective Cu loading for Cu^0^/WO_3_ NPs. They were trialed in H_2_ (g) releasing from DMAB at 60 ± 0.5 °C. The highest H_2_ generation rate was obtained for the catalyst sample of 4% wt. Cu. Consequently, the catalyst sample with 4.0% wt. Cu loaded was utilized for further catalytic reactions.

### 2.4. Recyclability test of Cu^0^/WO_3_ NPs catalyst

The catalytic performance of Cu^0^/WO_3_ NPs in subsequent runs of the reaction was tested. A typical reaction was started by preparing a suspension containing 100 mg Cu^2+^/WO_3_ (4.0% wt. Cu) and DMAB (60.11 mg, 100 mM) at 60.0 ± 0.5 °C. The procedure was repeated for the further run of the catalytic reaction, and performance of the catalyst was evaluated with determining turnover frequency.

### 2.5. Kinetic parameters of the reaction catalyzed by Cu^0^/WO_3_ NPs

Dehydrogenation reactions were repeated with 100 mg Cu^0^/WO_3_ (4.0% wt.) and a steady concentration of DMAB (100mM, 60.11 mg DMAB) in 10 mL toluene solution at various temperatures in order to get the activation parameters of the reaction.

### 2.6. Preparation and catalytic performance of Cu^0^/WO_3_ NPs

Catalytic reactions were carried out under inert gas atmosphere after vacuuming and purging of all glassware with nitrogen to remove any residue of oxygen and water. To prepare a stock solution, 153.02 mg copper bis(2-ethylhexanoate) was dissolved in 25.0 mL of toluene. For the preparation of the precatalyst, 3.75 mL (17.49 mM) of stock solution was transferred to a flask containing 100 mg of WO_3_ dissolved in 3.25 mL of fresh toluene. Next, DMAB dispersed in 3.0 mL toluene was added to the reaction medium. The slurry with a volume of 10.0 mL was stirred for an hour for impregnation of copper (II) ions on the surface of WO_3_. After an hour, DMAB was transferred to reaction medium by a gas-tight syringe. The reaction was followed until no more gas evolution.

## 3. Results and discussion

### 3.1. In situ preparation of Cu^0^ NPs supported on WO_3_

**Figure 1:**
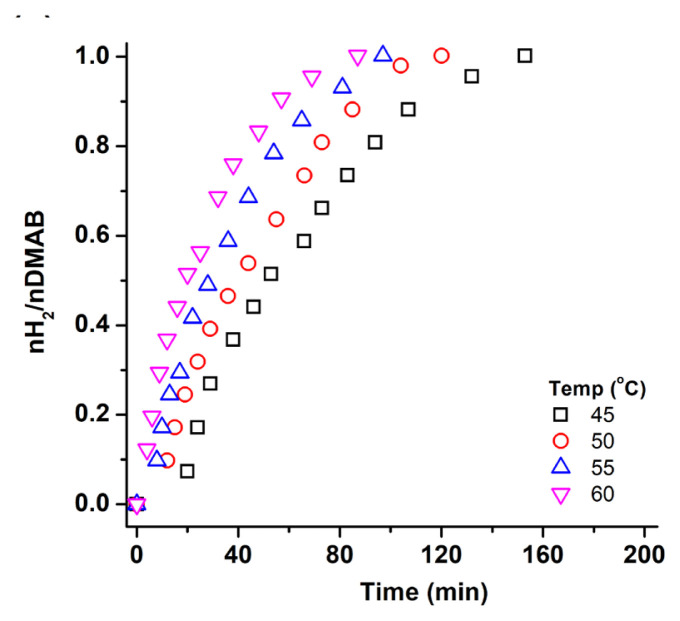
Plots of mol H_2_/mol DMAB versus time for H_2_ production reaction catalyzed by bare Cu^0^ NPs (red circle), WO_3_ supported Cu^0^ NPs (blue triangle) and bare WO_3_ (black square) at 60.0 ± 0.5 °C.

The reduction of Cu^2+^ ions on the surface of WO_3_ supporting material lead to active catalyst called Cu^0^/WO_3_ NPs. Cu^0^/WO_3_ NPs were tested as a catalyst in H_2_(g) generation from DMAB after the catalytic activity of bare Cu^2+^ ions were determined without using any stabilizer in dehydrogenation reaction. The comparison of H_2_ evolution from DMAB with bare Cu^2+^, bare WO_3_ and Cu^2+^/WO_3_ NPs can be seen in Figure 1. The same amount of Cu concentration was used in both experiments to compare each other. Although hydrogen evolution starts within a few minutes, bare Cu^0^ NPs have an initial 11.44 h^−1^ TOF value, lose their catalytic activity during the reaction course due to the aggregation of Cu^0^ NPs in the medium. The observation of bulk copper metal at the bottom of the reaction tube is also an evidence of the agglomeration of Cu^0^ NPs. The comparison of bare and WO_3_-supported Cu^0^ NPs indicates that adding 2-ethylhexanoate as only stabilizer in the reaction medium could not prevent the agglomeration of Cu^0^ NPs. On the other hand, Cu^0^/WO_3_ NPs exhibit important catalytic performance with 39 h^−1^ TOF value in the same catalytic reaction. It can be concluded that WO_3_ supporting material increases the catalytic activity of Cu^0^ NPs more than three times due to the large surface area. The complete H_2_ (g) production from DMAB was seen when the WO_3_-supported Cu^0^ NPs were used as an active catalyst. Therefore, WO_3_ was preferred as a stabilizer for Cu^0^ NPs. The comparison of bare Cu^0^ NPs, bare WO_3_, and WO_3_-supported Cu^0^ NPs in Figure 1 indicates that WO_3_ could prevent the aggregation of Cu^0^ NPs during the catalytic reaction.

**Figure 2:**
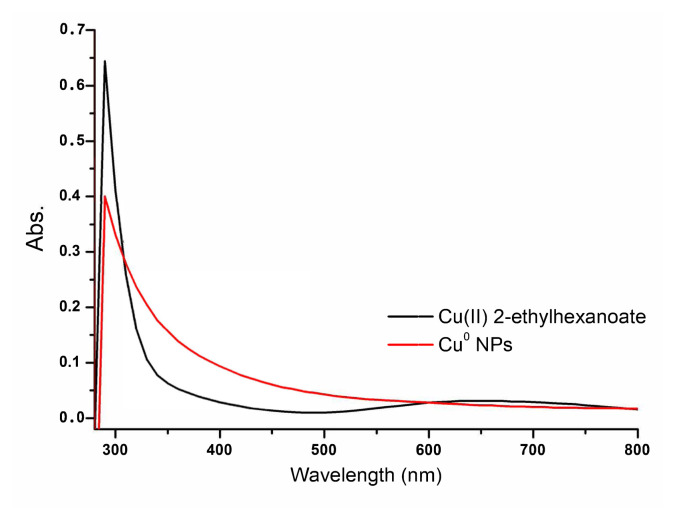
UV-Vis spectra of Cu precursor solution before and after addition of 100 mM DMAB 60.0 ± 0.5 °C.

The experiment was performed starting with 6.65 mM of copper(II) 2-ethylhexaonate, and 100 mg of WO_3_ in 10 mL of toluene. After addition of DMAB into the reaction medium, the color of the solution changes to dark brown. The traceable change in color of the solution allows following the reaction by UV-Vis. Figure 2 shows the UV-Vis spectrum of Cu^0^/WO_3_ NPs solution before and after transfer of DMAB into the reaction medium. UV-Vis spectrum of the beginning solution containing Cu^2+^ ions indicates a sharp absorption band at 290 nm and a broad band at around 651 nm. While the sharp absorption band at 290 nm is attributed to LMCT the other band at 651 nm shows the d-d transition of the copper salt used as a precursor in this study [33]. The new spectrum obtained after the reaction was completed demonstrates that the bands disappeared and a new band at 289 nm was observed. This new band that appeared at 289 nm shows typical Mie scattering for Cu^0^ NPs, which implies the formation of Cu^0^ during the catalytic reaction [34].

**Figure 3:**
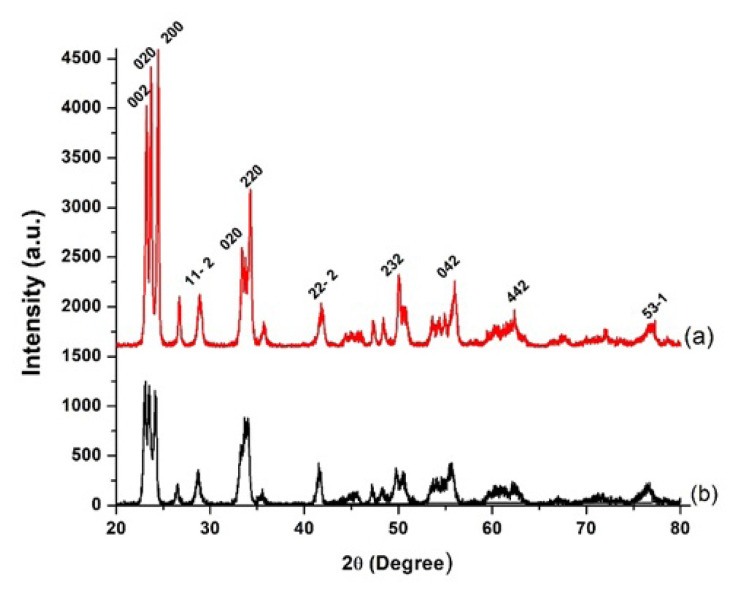
Powder p-XRD patterns of a) unloaded WO_3_ nanopowder (red line), b) WO_3_ supported Cu0 NPs (Cu^0^/WO_3_, 4.0% Cu wt.).

The isolated Cu^0^/WO_3_ NPs were identified by modern analytical techniques. Figure 3 displays the powder X-ray diffraction patterns of bare WO_3_ and WO_3_-supported Cu^0^ NPs. Both samples show the same diffraction peaks that belong to WO_3_ (ICDD Card No: 43-1035), which approves that: (i) the traceable peak would be attributed to Cu has not been seen, probably due to lower metal loading and (ii) the lattice and crystallinity of WO_3_ are not affected by reduction of Cu^2+^ ions to Cu^0^ on the surface of powder WO_3_ supporting material. Moreover, the positions of diffraction peaks of WO_3_ maintain their initial positions after Cu loading on the surface of WO_3_ sample. As a result, it can be expressed that Cu loading does not alter the infrastructure of support.

**Figure 4:**
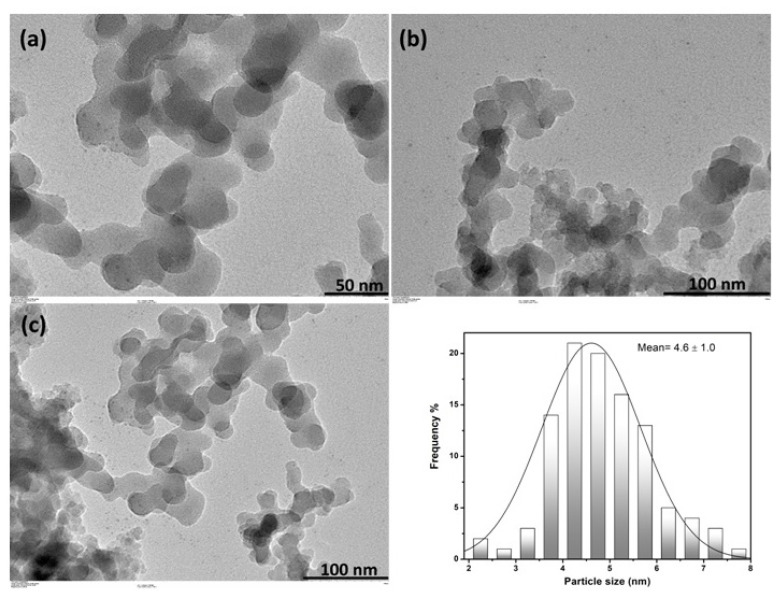
(a,b,c) TEM images of Cu^0^/WO_3_ with Cu 4.0 % loading after dehydrogenation of DMAB in different magnifications 50, 100 nm d) The histogram of Cu^0^ NPs.

TEM images of Cu^0^/WO_3_ NPs samples with 4.0% Cu loaded are shown in Figure 4. In more details, TEM images with different magnification explain that Cu^0^ NPs are well-dispersed on the surface of WO_3_. The particle size histogram was constructed by counting more than 100 nontouching particles, and the mean diameter of Cu^0^ NPs was found as (4.6 ± 1.0) nm.

**Figure 5:**
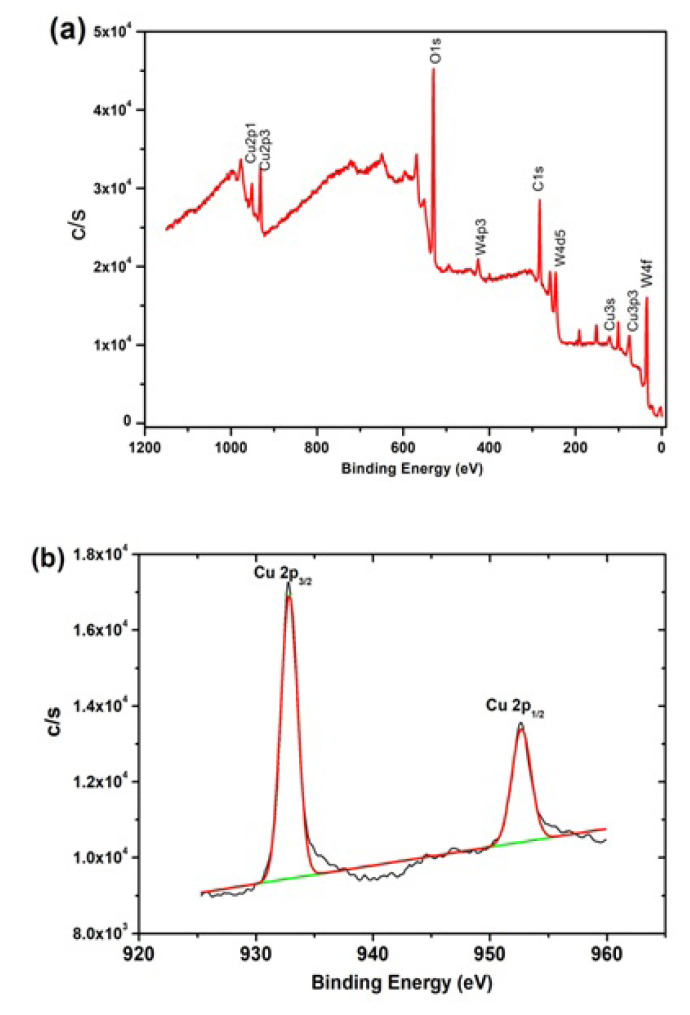
(a) (XPS) survey scan of Cu^0^/WO_3_ catalyst sample (Cu 4.0% wt.), (b) Detailed analysis of Cu 2p bands.

XPS analysis offers important insights for the chemical state information of copper in the catalyst sample from the surface of Cu^0^/WO_3_. The survey analysis of Cu^0^/WO_3_ NPs sample in Figure 5 displays the copper element with the framework elements of WO_3_. High-resolution spectrum of Cu 2p bands indicates that two prominent peaks at 932.8 and 952.7 eV belong to metallic Cu 2p_3/2_ and Cu2p_1/2_, respectively [35,36]. XPS analysis is evidence of reduction of Cu^2+^ ions used as a precursor and existence of metallic Cu as a Cu^0^ form in the catalyst sample.

### 3.2. Catalytic performance of Cu^0^/WO_3_ NPs

**Figure 6:**
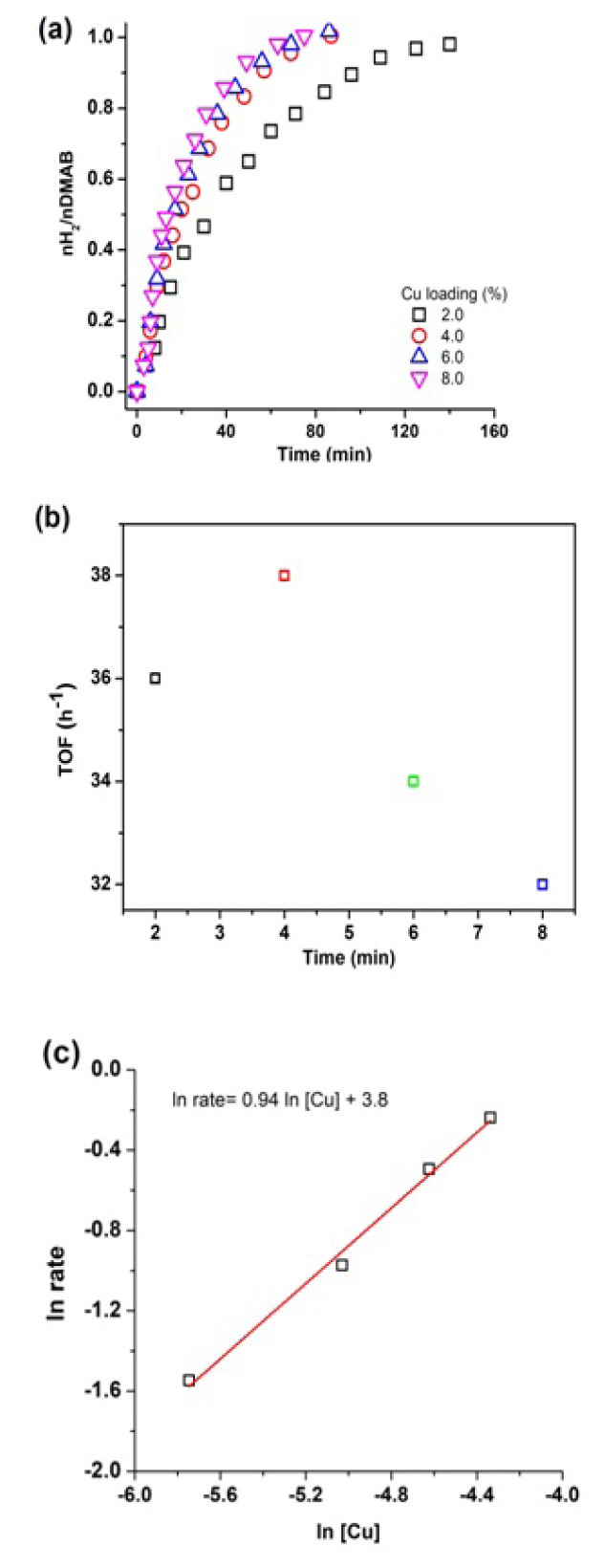
(a) Plots of mol H_2_/mol DMAB versus time for hydrogen generation from dehydrogenation of 100 mM DMAB in different copper loadings (2%, 4%, 6%, 8% wt. Cu), (b) TOF values versus Cu loadings of catalyst Cu^0^/WO_3_, c) Logarithmic plot of rate versus metal concentration.

Firstly, the catalytic activity of bare WO_3_ sample was tested in H_2_ generation from DMAB starting with 100 mg of WO_3_ in 10.0 mL of toluene. The reaction was followed for at least one hour. It is obviously clear that WO_3_ is catalytically inert in its bare form for the dehydrogenation reaction. Next, Cu-loaded WO_3_ samples with different percentages of Cu were prepared and trialed in dehydrogenation of DMAB. Figure 6a shows the H_2_(g) evolution graph from DMAB catalyzed by Cu^0^/WO_3_ NPs with different Cu loading in the range 2%–8% wt. Cu. H_2_ evolution starts within a few minutes after transfer of DMAB substrate into reaction mixture and continue until 1.0 equiv gas evolved from the catalytic reaction. The sample with 4.0% wt. Cu loaded found as the most active catalyst in H_2_ (g) production from DMAB. The H_2_ generation rate of the catalyst was calculated as 39 h^−1^ for Cu^0^/WO_3_ NPs with 4.0% wt. Cu. The relationship between the catalytic activity of the catalyst and copper loadings in the sample can be seen in Figure 6b. A volcano-shaped variation was seen as the copper loading increased most probably due to pile aggregation of Cu^0^ NPs on the surface of supporting material during the reaction. The further copper loadings lead to a decrease in surface area of the supporting material and failure to reach active sites of the catalyst. Therefore, the Cu^0^/WO_3_ samples with a ratio of 4.0% wt. Cu were used as the optimum ratio for all further experiments in this work. In order to determine the effect of catalyst concentration, the rate of the reaction was determined from each graph. As shown in Figure 6c, a straight line with a slope of 0.94 indicates that the catalytic reaction is a first-order reaction with respect to concentration of copper.

**Figure 7:**
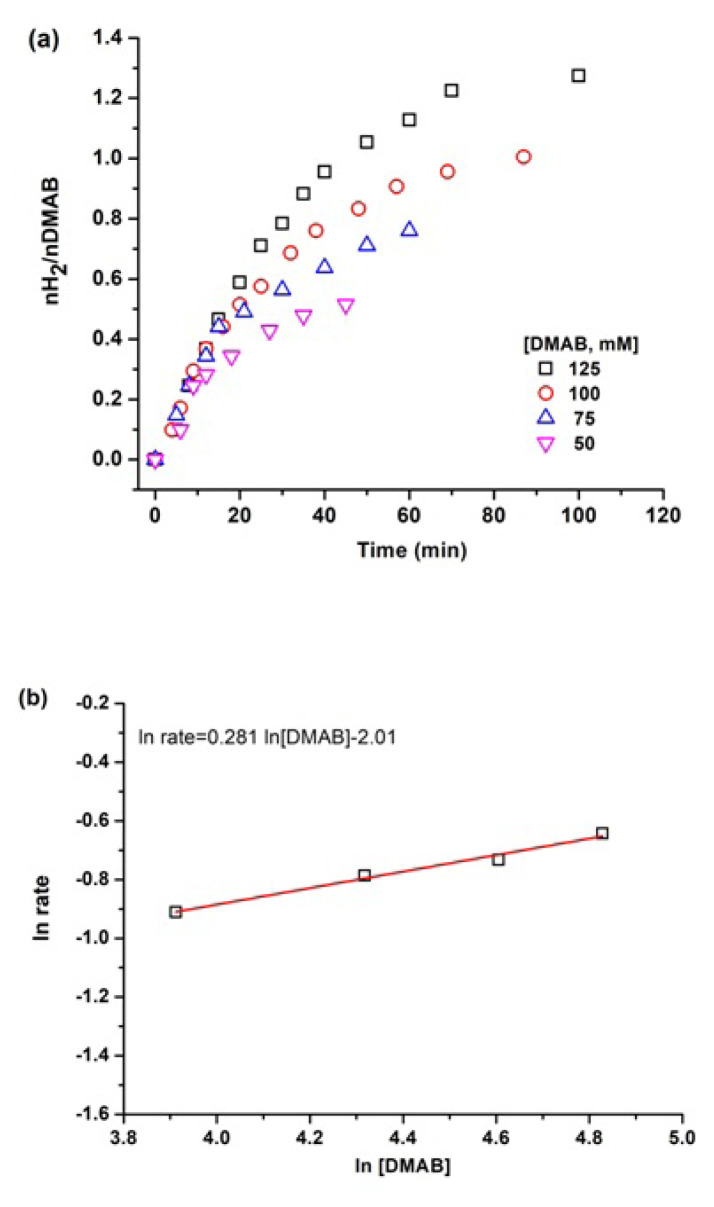
a) The plot of hydrogen generation graph with different substrate concentration in the range of 50–125 mM while keeping the catalyst concentration 6.65 mM for each experiment, b) ln rate vs ln DMAB graph.

Similar experiments were performed to determine the substrate effect on the rate of dehydrogenation reaction of DMAB while keeping the catalyst concentration constant at 6.65 mM Cu (Figure 7a). It can be clearly seen from the slope of line in Figure 7b that hydrogen generation rate from the catalytic dehydrogenation of DMAB is actually independent of DMAB concentration. Thus, dehydrogenation of DMAB catalyzed by Cu^0^/WO_3_ NPs is approximately zero order with respect to the substrate concentration. Thus, the rate law of dehydrogenation reaction can be given as Eq. (2):


(2) $$\text{Rate}={\text{k}}^{\text{app}}{\left[\text{Cu}\right]}^{\text{a}}{\left[\text{DMAB}\right]}^{\text{b}},$$

where a and b were found as 0.94 and 0.28, respectively.

**Figure 8:**
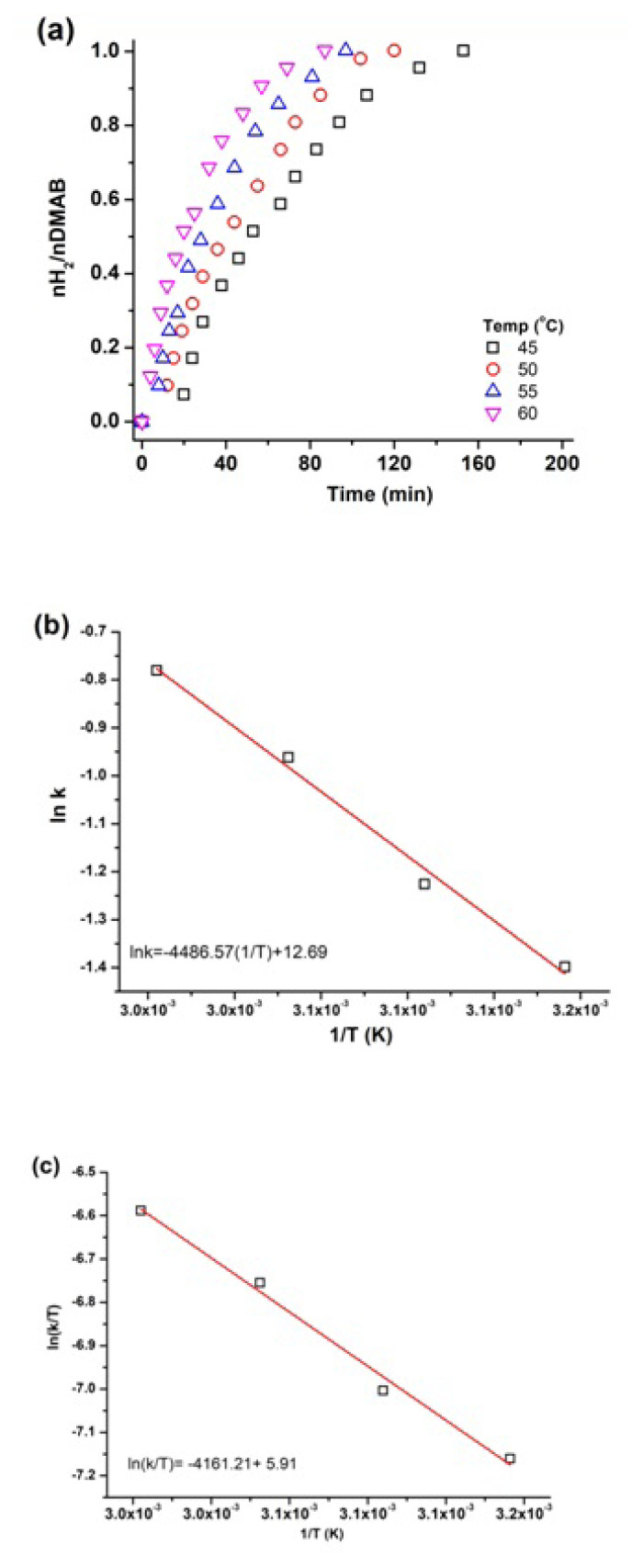
a) H_2_ evolution graph of 100 mM DMAB catalyzed by Cu^0^/WO_3_ NPs (%4.0 Cu wt) at various temperatures (45–60 °C), b) Arrhenius, and c) Eyring plot.

The dehydrogenation experiments were also repeated to determine the activation parameters of the reaction. The catalytic performance of WO_3_ supported Cu0 NPs at different temperatures can be seen in Figure 8a. The rate constant for each plot was calculated from the experimental data and utilized to draw the Arrhenius and Eyring plots [37,38]. The slope of the Arrhenius (Figure 8b) and Eyring plots (Figure 8c) gave the activation energy and entropy of the catalytic reactions as *E*_a_ =37 ± 2 kJ mol^−1^, ΔH^#^=35 ± 2 kJmol^−1^ and ΔS^#^=−148 ± 2 JK^−1^mol^−1^, respectively, which are comparable to that previously reported in the literature [39]. Table indicates the catalyst used in dehydrogenation of DMAB with TOF values and some reaction parameters.

**Figure 9:**
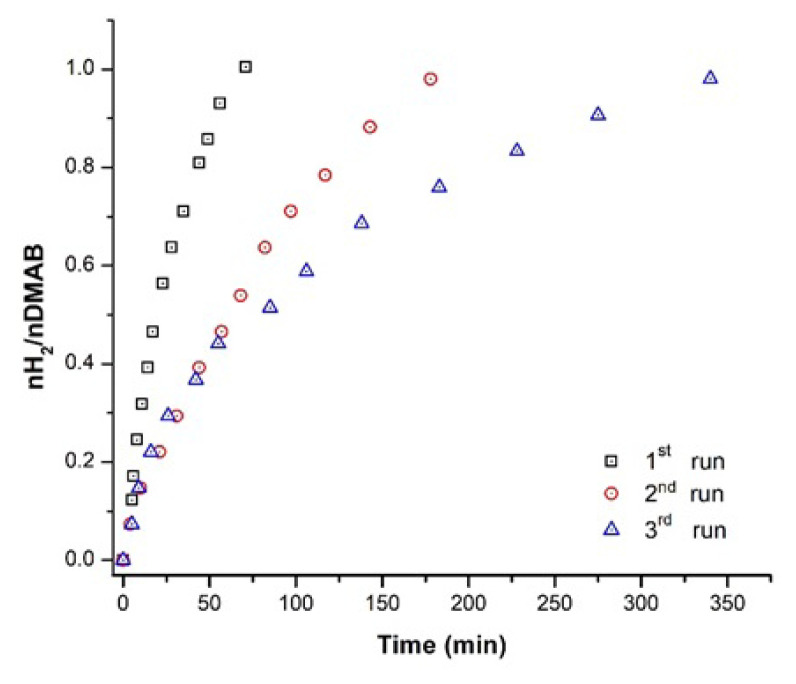
Dehydrogenation plots of 100 mM DMAB catalyzed by Cu^0^/WO_3_ NPs with 4.0% wt. Cu. in the subsequent runs.

In order to explain the catalytic performance of Cu^0^/WO_3_ NPs in subsequent runs, recyclability tests were also executed. The system was preserved without any alterations after first run of the catalytic dehydrogenation and the same amount of dimethylamine borane was added into the medium. H_2_ (g) generation was followed for the following runs of the catalytic reaction. It was seen in Figure 9, Cu^0^/WO_3_ NPs catalyst remains catalytically active in H_2_(g) generation from DMAB more than three cycles of the dehydrogenation reactions. The decrease in catalytic activity of the Cu^0^/WO_3_ NPs attributed to the fact that addition of more DMAB in further runs causes the agglomeration of Cu^0^ NPs on the surface of the supporting material. The other reason for the decrement of catalytic activity of the reaction is related to the accumulation of the side product of the DMAB.

**Figure 10:**
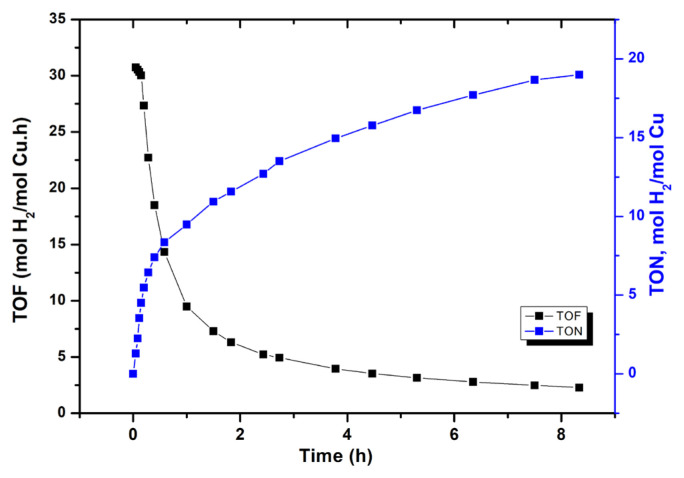
Catalytic lifetime of Cu^0^/WO_3_ NPs catalyst in dehydrogenation of DMAB using 6.54 mM Cu in 10.0 mL toluene solution including 100 mg of WO_3_ sample and 250.0 mM DMAB at 60.0 ± 0.5 °C.

The catalytic lifetime of WO_3_ supported Cu^0^ NPs in H_2_ releasing from DMAB was determined by measuring the total turnover number (TTON). Lifetime of the catalyst was performed starting with 0.0654 mmol Cu in 10.0 mL toluene solution including 100 mg WO_3_ sample and 250.0 mM DMAB at 60.0 ± 0.5 °C (Figure 10). TTON of the catalyst was measured by adding more DMAB after each complete conversion. The reaction was followed until no more hydrogen evolution was observed. Initial TOF value of 30 h^−1^ was obtained for Cu^0^/WO_3_ NPs in lifetime experiment performed at 60.0 ± 0.5 °C. The decrease in catalytic activity within hours indicating the deactivation of the catalyst

**Figure 11:**
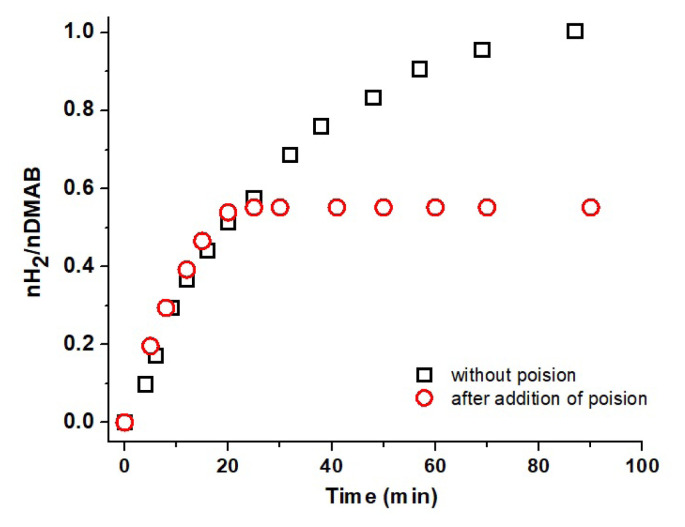
Plots of nH_2_/nDMAB vs time for dehydrogenation of DMAB catalyzed by 4.0% wt. Cu/WO3 NPs before and after addition of 0.20 eqiv. of CS_2_ at 60.0 ± 0.5 °C.

Poisoning experiment was also carried out to test whether the catalytic dehydrogenation reaction of DMAB is homogeneous or heterogeneous. The poisoning test for Cu^0^/WO_3_ NPs catalyst was performed with CS_2_ (0.2 eqiv. per mole of catalyst used). After evolution of 50% H_2_ from the reaction, 0.2 eqiv. CS_2_ was transferred to reaction flask via gas tight syringe. Hydrogen evolution was suddenly ceased and no more H_2_ evolved from the reaction. It was clearly seen in Figure 11 that Cu^0^ NPs supported on surface of WO_3_ act as a heterogeneous catalyst during the dehydrogenation of DMAB.

## 4. Conclusion

The main outputs of the study can be summarized as: Cu^0^ NPs obtained from the reduction of Cu(II) ions show a catalytic activity in dehydrogenation of DMAB. However, they need a stabilizer to prevent the agglomeration during the reaction course. WO_3_ was used as stabilizer for Cu^0^ NPs in this study. The comparison of bare and WO_3_-supported Cu^0^ NPs indicates that usage of supporting material increase the catalytic activity of Cu^0^ NPs more than three times in H_2_ releasing reaction of DMAB at 60.0 ± 0.5 °C.

1. Isolated Cu^0^/WO_3_ NPs were characterized by advanced techniques (XRD, TEM, XPS, and UV-Vis spectroscopy). The results disclosed that Cu^0^ NPs with a mean particle size as 4.6 ± 1.0 nm could be stabilized on surface of WO_3_.
2. The order of reaction found as one was calculated from the results of kinetic studies with respect to catalyst concentration.
3. Recyclability tests clarified that Cu^0^/WO_3_ NPs lose their initial activity in the further catalytic cycles. The decreased in activity of the Cu^0^/WO_3_ catalyst for the sequent runs may be due to the aggregation of Cu^0^ NPs on the surface of tungsten(VI) oxide.
4. Relatively high activity, low cost, and the simple preparation of Cu0/WO3 catalyst make the Cu^0^ NPs possible nominee to be utilized as attractive catalysts in developing an efficacious H_2_(g) production using DMAB as solid hydrogen storage materials.

## Figures and Tables

**Table:** Comparison of the TOF values and some reaction parameters of the catalysts used in H2 generation from DMAB.

					Equiv. H_2_				
Entry	Precatalyst/catalyst	Solvent	T (°C)	Time (h)	per mole of DMAB	Particle size(nm)	TOF (h^1^)	E_a_ (kJ/mol)	Ref.
**1**	[Ru(H)(PMe_3_)(N(C_2_H_4_P*^i^*Pr_2_)_2_]	THF	25	28	1.0	-	1.5	-	[40]
**2**	[Rh(1,5-cod)(μ-Cl)]_2_	Toluene	25	8	1.0	-	12.4	-	[10]
**3**	Cp_2_Ti	Toluene	20	4	1.0	-	12.3	-	[41]
**4**	[Ir(1,5-cod)(μ-Cl)]_2_	Toluene	25	136	0.95	-	0.7	-	[10]
**5**	Rh(0)/[Noct_4_]Cl	THF	25	6	0.9	2.0	8.2	-	[42]
**6**	RhCl_3_	Toluene	25	23	0.9	-	7.9	-	[10]
**7**	[RhCl(PHCy_2_)_3_]	Toluene	25	19	1.0	-	2.6	-	[47]
**8**	Rh(0) Nanoclusters	Toluene	25	2.5	1.0	1.9	60	34	[22]
**9**	Pd(0)/MOF	Toluene	25	6	1.0	4.3	75	173.5	[43]
**10**	Pt(0)NPs/AA	THF	25	0.6	1.0	3.3	15	64	[44]
**11**	Pt(0)/TBA	THF	25	1	1.0	3.9	31	46.7	[45]
**12**	[PtH(I*^t^*Bu)(I*^t^*Bu)]	THF	25	-	1.0	-	-	-	[46]
**13**	Pt(0)/DPA@GO	THF	25	1	1.0	3.6	35	42	[47]
**14**	OAm-stabilized Ru(0)NPs	Toluene	25	1.5	1.0	1.8	137	29	[48]
**15**	Ru(0)APTS	THF	25	2	1.0	1.7	55	61	[49]
**16**	OAm-stabilized Cu(0) NPs	Toluene	50	1.3	1.0	3.5	158	19	[23]
**17**	Cu(0)/CeO_2_	Toluene	60	0.6	1.0	3.1	40	76	[46]
**18**	Pd(0)/Al_2_O_3_	Toluene	25	1.3	1.0	7.1	73	36	[29]
**19**	Ru(0)/CeO_2_	Toluene	60	1	1.0	1.8	812	62	[25]
**20**	Cu^0^/WO_3_	Toluene	60	1.5	1.0	4.6	39	37	This Study
